# Creativity and Artificial Intelligence—A Student Perspective

**DOI:** 10.3390/jintelligence10030065

**Published:** 2022-09-06

**Authors:** Rebecca Marrone, Victoria Taddeo, Gillian Hill

**Affiliations:** 1 The Centre for Change and Complexity in Learning, The University of South Australia, Adelaide 5000, Australia; 2 Centre for Research in Expertise Acquisition, Training and Excellence, School of Psychology, University of Buckingham, Buckingham MK18 1EG, UK

**Keywords:** creativity, artificial intelligence, student attitudes

## Abstract

Creativity is a core 21st-century skill taught globally in education systems. As Artificial Intelligence (AI) is being implemented in classrooms worldwide, a key question is proposed: how do students perceive AI and creativity? Twelve focus groups and eight one-on-one interviews were conducted with secondary school-aged students after they received training in both creativity and AI over eight weeks. An analysis of the interviews highlights that the students view the relationship between AI and creativity as four key concepts: social, affective, technological and learning factors. The students with a higher self-reported understanding of AI reported more positive thoughts about integrating AI into their classrooms. The students with a low understanding of AI tended to be fearful of AI. Most of the students indicated a thorough understanding of creativity and reported that AI could never match human creativity. The implications of the results are presented, along with recommendations for the future, to ensure AI can be effectively integrated into classrooms.

## 1. Introduction

There is a strong consensus that creativity is a crucial 21st-century competency. Education systems report the importance of creativity (Patston et al. 2021). Similarly, Artificial Intelligence (AI) is significantly impacting a growing number of fields, including education (Gabriel et al. 2022). Globally, education systems are developing strategic plans to embed AI in classrooms adequately (see Singapore, Estonia, Australia, New Zealand, and Scotland, to name a few) (Gabriel et al. 2022). Whilst the importance of both creativity and AI are well established, less is known about how students perceive and value the relationship between AI and creativity. This paper will explore how students perceive AI and creativity, and endeavour to ensure that education systems support the development of both competencies. 

### 1.1. Artificial Intelligence in Education

Artificial Intelligence (AI) is a branch of computer science that uses algorithms and machine learning techniques to replicate or simulate human intelligence (Helm et al. 2020). There are three types of AI: narrow AI, general AI, and Artificial Superintelligence. Narrow AI is the most common and realized form of AI to date. It is very goal-orientated and uses machine learning techniques to achieve one specific goal or task (e.g., image and facial recognition, Siri/Alexa). General (or deep) AI is AI that is deemed on par with human capabilities (e.g., AI that can discern the needs and emotions of other intelligent beings). Thirdly, Artificial Superintelligence is AI that is more capable than humans (similar to a sci-fi movie portrayal of AI that supersedes humans in every regard) (Hassani et al. 2020).

Within the education context, artificial intelligence development will likely remain in the form of narrow AI. Current educational technologies include speech semantic recognition, image recognition, Augmented Reality/Virtual Reality, machine learning, brain neuroscience, quantum computing, blockchain, et cetera. These technologies are rapidly being integrated within classrooms. An ever-increasing number of artificial intelligence education products are being applied to K-12 education (Yufeia et al. 2020). Literature studies show that artificial intelligence technology in education has been used in at least 10 aspects: “the (i) automatic grading system, (ii) interval reminder, (iii) teacher’s feedback, (iv) virtual teachers, (v) personalized learning, (vi) adaptive learning, (vii) augmented reality/virtual reality, (viii) accurate reading, (ix) intelligent campus, and (x) distance learning” (Yufeia et al. 2020, p. 550).

The Artificial Intelligence in Education (AIED) community emphasises the creation of systems that are as effective as one-on-one human tutoring (VanLehn 2011). Over the last 25 years, there have been significant advances toward achieving that goal. However, by enforcing the human tutor/teacher as the gold standard, a typical example of AIED practices has often included a student working with a computer to solve step-based problems focused on domain-level knowledge in subjects such as science and mathematics (Trilling and Fadel 2009). However, this example does not consider the recent developments in education practices and theories, including introducing 21st-century competencies. The 21st-century competency approach to education emphasises the value of general skills and competencies such as creativity. Today’s classrooms strive to incorporate authentic practices using real-world problems in collaborative learning settings. To maintain its relevance and increase its impact, the field of AIED has to adapt to these changes. 

### 1.2. What Does Creativity in an AI Classroom Look Like?

Boden (1998), in her paper, suggests that AI techniques can be used to enhance creativity in three ways: ‘by producing novel combinations of familiar ideas; by exploring the potential of conceptual spaces; and by making transformations that enable the generation of previously impossible ideas’ (p. 1). While there have been attempts to combine the fields of AI and creativity, and to define them through the emerging field of computational creativity, it has often ended in confusion. Computational creativity (CC) (also known as artificial creativity or creative computation) places AI/computers at the centre of creativity (Colton and Wiggins 2012). Computational creativity is underpinned by Rhodes’ 4P’s of creativity theory, which emphasises that creativity is an interaction between four factors: process, person, product, and press (environment) (Rhodes 1961). While all four factors are crucial for human creativity, Cropley et al. (2021) have suggested that only two factors are important for human and artificial creativity: process (i.e., cognition), and product (i.e., outcome). Creative products are measured by novelty and effectiveness (Cropley and Cropley 2012; Cropley and Kaufman 2012), where novelty refers to a new or original idea or concept, and effectiveness is the ability of the product or solution to achieve its desired result. Process is defined as the cognitive mechanisms of creativity and is key to understanding what artificial intelligence can offer to develop novel and effective solutions to problems. Therefore, to encourage the use of creativity and AI, educators should consider the process by which creativity has unfolded and/or the product of the creative endeavour.

There is emerging research on assessing the creative product using AI-based methodologies. Cropley and Marrone (2021) demonstrate how AI can successfully assess figural creativity using convolutional neural networks. Beaty and Johnson (2021), and Olson et al. (2021) also demonstrate the use of latent semantic analysis to assess the creativity of student responses to a traditional alternate uses task. While this is a growing field, this research focuses more on the outcome or product of creativity and less on the process. 

### 1.3. The Process of Creativity and AI

Students should be aware of how AI can support their creativity and learning. Modern education favours problem-solving-based pedagogies, which emphasise the importance of fostering children’s ability to think creatively. However, considerable research supports the existence of a creativity slump in younger children across subjects (Torrance 1968; Tubb et al. 2020). One proposal for this slump is an overly structured school curriculum and a lack of play-based learning activities in educational practices (Alves-Oliveira et al. 2017). Emerging research shows how AI can support skills often associated with creativity, such as curiosity (Gordon et al. 2015), grit, persistence, and attentiveness (Belpaeme et al. 2018). The ability of AI to support creativity is also being explored. Kafai and Burke (2014), in their study, report that the purpose of AI in education is to encourage and support skills such as problem-solving and creativity through collaboration with AI, rather than simply acquiring knowledge in the specific domain. The paper suggests that AI can help creativity unfold and is therefore related to the process through which creativity occurs. Furthermore, Ryu and Han (2018) studied Korean school teachers’ perceptions of AI in education and report that teachers with experience in leadership recognized that AI would help to improve creativity. Therefore, it is proposed that AI in education may address some of the main concerns associated with the creativity slump, particularly an emphasis on the creative process. This may help improve creative thinking in students and comfortability using AI, and to adequately prepare students to enter the modern workforce. 

To successfully combine and integrate AI and creativity, we must better understand how students perceive the relationship between the two concepts. To understand this perception, we should also situate AI with other predominant creativity theories, including the 4C model of creativity.

### 1.4. A 4C Approach to AI

Creativity and AI in an educational context can be viewed through a 4C model (Kaufman and Beghetto 2009). Mini-c or ‘personal creativity’ embodies the personal (Runco 1996; Vygotsky 2004) and developmental (Cohen 1989) aspects of creativity. Mini-c relates to subjective self-discoveries that are creative to the individual involved and not necessarily others. An example may be an individual making a slight variation on a well-known recipe. Little-c is also called ‘everyday creativity’ and refers to something other people recognise as creative, such as generating a new recipe. Pro-c or ‘professional creativity’ is defined as becoming an expert in any field or discipline. An example may be the chef, Gordon Ramsey. Big-C or ‘legendary creativity’ is defined as eminent creativity and will be remembered for centuries. An example may be August Escoffier, who is credited as the founder of modern cuisine and has dramatically altered the field of cooking (Beghetto et al. 2016).

Most obviously, AI can support creativity at the pro-c and potentially Big-C levels, as it can help extend expert knowledge in specific domains. Less obvious is how AI can support mini-c and little-c contributions. At the mini-c and little-c levels, the creative output is not as crucial as the self-discovery that occurs through the creative process. It is therefore essential to develop both an appreciation and understanding of when and where AI is most valuable, that is, in what narrow domains does AI best suit education, and how can AI be used to encourage mini-c and little-c contributions?

This research will investigate how students perceive AI and creativity, and the relationship between the two. We expect insights to highlight how AI can be designed to support creativity in the classroom.

## 2. Materials and Methods

### 2.1. Participants

Eighty secondary school students from four South Australian schools (mean age 15) participated in an eight-week programme. Students were tasked with the challenge of: ‘How do we sustain life on Mars?’ Sixty students completed this task as part of their regular science class. Twenty students completed this task as an extracurricular after-school programme. The programme’s content was identical, irrespective of whether the student participated in their regular science class or as an extracurricular activity. The same staff conducted both versions.

### 2.2. Method

Grounded theory (GT) is a structured yet flexible methodology that is appropriate when little is known about a phenomenon (Chun Tie et al. 2019). Grounded theory investigates the experience of people and their responses and reactions and generates a theory. A defining characteristic of GT is that it aims to generate a theory that is grounded in the data. Considering there is minimal research on student perceptions of AI and creativity, this methodology was chosen.

### 2.3. Context

The students explored a variety of sub-problems related to their task; however, one task was around designing and building a Mars Rover. Those who engaged as part of their science class worked in groups of 4–5 students, and each team spent one week (four × 50-min lessons) engaging solely with artificial intelligence and building their Rover. For the other seven weeks, students engaged with the AI system, once a lesson for approximately 10 min each time (40 min per week over seven weeks). The students who engaged in the extracurricular version of this programme also were in groups of 4–5 and engaged with the AI system for six hours over a one-day, in-person event. The other lessons were hosted on Zoom and did not involve AI. The students physically built a Mars Rover using Fischer Technik kits and then engaged with an AI-based vision analytics tool to receive feedback on their build. Whilst the technology behind the vision analytics tool has been created by individuals at the pro-c level, its application in the classroom was created to elicit mini-c or little-c creativity in students. This is because the students use the system to get specific and targeted feedback on every step of their build. The students can then use this information to decide if the AI is helping them achieve their goals of creating the Rover. Once students had built their Rover, the vision analytics system could scan it and upload it into a 3D virtual environment, where students could drive their Rover on Mars. Here they learnt about planetary factors, such as gravity, and terrain. 

This was an open-designed task with no instructions, and students were instructed to be creative with their choices and designs. They received creativity training, specifically: “What is creativity and what is it not?”. 

### 2.4. Data Analysis

Twelve focus groups were conducted with the students engaged with this project in their regular science lessons. Eight one-on-one interviews were conducted with those students who participated in this programme as an extracurricular programme. The questions asked to all the students were the same, regardless of whether they engaged in their class or as an extracurricular activity. The interviews were framed around how students perceive both AI and creativity. See Appendix A for the interview questions. A content analysis methodology was used to analyse the meaning of the participants’ narratives. Fraenkel et al. (2006) define content analysis as ‘a technique that enables researchers to study human behaviour in an indirect way, through an analysis of their communications (p. 483). The purpose of content analysis is to explore participants’ verbal communication and social behaviour without influencing it. Content analysis allows a researcher to interpret what is being communicated, why it is being communicated, and with what effects (Wagenaar and Babbie 2004). An objective codification process characterises content analysis and involves placing coded data into key categories and more abstract concepts.

One conceptualisation of creativity and AI that emerged from the students’ remarks was labelled ‘Social Factors’. Typical categories defining the concept were ‘conversation and lack of awareness’, ‘student interest’ and ‘social intelligence/social skills’. Another different conceptualisation identified in the content units was ‘affective’. Typical categories defining this concept were ‘comfortable with AI’ and ‘not comfortable with AI’. A different kind of conceptualisation was observed in the cognitive view expressed by some of the students interviewed. This led to the concept ‘Technological Factors’. The typical categories here were ‘access and use of AI’, ‘technology focused’, ‘robotics’, and ‘computers’. The final concept was labelled as ‘Learning Factors’. The typical categories related to the student’s current school environment were ‘AI provides a learning aid’, and ‘creativity takes time’. These concepts are shown in Appendix B, along with the content units from which they were derived, and the categories defined by these content units.

## 3. Results and Discussion

This study aimed to understand how the students view the relationship between AI and creativity. This topic was addressed through a content analysis interpretation of the students’ responses to key questions. The results highlight that the students in the study understood the relationship between AI and creativity as four fundamental concepts: social, affective, technological and learning factors.

### 3.1. Social Factors

The results from the interviews suggest that secondary school students in Australia hold opinions that AI can negatively impact their social skills. The AI facilitators/barriers category tended to include negative views and perceptions of AI. Previous research notes that AI will drive us into roles that require more social skills and typically encourage these social-based roles (Deming 2017; Makridakis 2017). However, the students believed that AI would negatively impact their social skills. Comments such as ‘AI can make people lack ‘social-wise’. AI can make social intelligence weaken a little bit, which can affect them (students), and another comment: ‘Well, if we’re talking about robots and such for computers and phones and digital media social media, that kind of stuff…it’s taking away from people’s social lives, and they’re just more concerned about having a digital platform to present themselves on, rather than focusing on presenting themselves in the physical world.’. One student reported that getting AI to become ‘a mainstream thing so everyone can speak to everyone on it, so we can ask whole communities and get out with a lot of people’ was essential to changing the conversations about AI. These somewhat negative perceptions may hinder students’ willingness to adopt AI technologies in their classrooms. Chai et al. (2021) demonstrate that the intention to learn AI in primary school students is influenced by the students’ perception of the use of AI for social good. Furthermore, Chai et al. (2020) highlight that students perceive the purpose of learning of AI for social good as the most powerful predictor for their behavioural intention to continue learning AI. The students also reported that AI will never work in fields where human skills are required for problem-solving. When asked whether AI can match human skills, one focus group reported that the father of a participant in this group was a pilot. They mentioned that it was crucial AI never entered the cockpit as humans should be tasked with solving a complex problem like flying a plane. Interestingly, every member of this group agreed and seemed apparently unaware of the level of technology that is associated when flying. This represents a gap in student understanding of how AI can be used to assist humans. The students in this group failed to see the value of AI as a teammate and solely viewed this role as a human skill. Further emphasis should be placed on educating students on the role of human–AI teaming, and that AI can support humans, even in seemingly social or complex situations. The belief that AI can negatively hinder their social skills also represents an opportunity to demonstrate how AI can benefit social skills and enhance connections across communities. 

### 3.2. Affective Factors

Students reported various affective responses to AI. Those students who verbally reported feeling more familiar with AI also reported feeling more comfortable using AI technologies. However, the students who said they were not sure what AI was, also said they felt less comfortable defining AI, as well as integrating it into their classrooms. This finding is supported by both Chiu (2017), and Teo and Tan (2012). These authors highlight that a positive attitude towards technology can explain one’s intention of using the technology. One student reported feeling comfortable because he had ‘all the safety programmes on it (his computer)’, so he reported having trust in his AI systems. Another student responded, ‘depends on the type of AI, so, I guess computers and programming and telling a computer instructions’. When prompted, they reported they wouldn’t feel as comfortable using ‘robots and machines’. This transparency in the AI system relates to an increase in trust in the AI. This is in line with previous research that transparency and the avoidance of ‘black box’ suggestions can foster AI adoption. This is referred to as explainable AI (Lundberg et al. 2020). 

### 3.3. Technological Factors

Interestingly, the majority of the students’ perceptions of AI were related to technological factors. Categories such as advanced technology, automation, coding/programming, futuristic, not human and robots, all had a lot in common. Students typically thought of AI as robots or computer-based, as this is how they interact with AI in their daily lives. These comments can be interpreted as the students possessing quite a limited view of AI applications, and they all struggled to move beyond the idea that AI is more than robots and computers. Several students felt that AI was a ‘futuristic’ phenomenon and was not as impactful in their current lives. All students reported that AI, to them, included some form of robotics. Chiu et al. (2021) and Chiu and Chai (2020) suggest that students should learn about AI by referring to real-life applications that they are likely to encounter in their daily experiences.

When asked if AI can ever match human creativity, students reported that, despite AI being technically superior to humans, human creativity will always be a uniquely human trait that should be fostered. One student commented, ‘Basically, most things in artificial intelligence are made by humans so, unless we actually create a robot which can be a human, it probably won’t be able to match the creativity of humans.’. The students who did believe that AI could match human creativity suggested that ‘maybe over time, when technology gets a lot more advanced, I think that it would be eventually possible to be as creative as humans’. Thus, they didn’t think AI could currently match human creativity but may do so in the future. When asked ‘do you think AI could ever match human creativity?’ One student made a very interesting comment. She said, ‘Yes, kind of. It’s a very interesting question. I think it can spark creativity. I don’t know if AI itself (can be creative). I don’t know if a robot can be creative because, in order for a robot to be creative, someone has had to create the robot and give it its creativity as such, so I don’t know if they can be creative themselves, but I think they can spark creativity.’. Therefore, they view AI as a way to facilitate or ‘spark’ creativity. Based on these comments, it is suggested that AI should be used to enhance creativity. Markauskaite et al. (2022), in their recent paper, demonstrate how AI can be used to support creativity across different age groups. The authors polylogue provides concrete suggestions based on a 4C theory of creativity approach on how and where AI can be used to enhance creativity, particularly for students.

### 3.4. Learning Factors

The most frequent and mentioned categories are related to the concept of learning factors. The students reported a positive view of AI and that it can support them to access information more efficiently; it can promote global connections, support their ideas, and aid learning. The students also reported that the benefits of creativity include time management and increasing their novel ideas. However, students also reported that their current school environments sometimes negatively impact their ability to exhibit creativity. Unsurprisingly, students mentioned not having enough time to be creative and that assignments were not designed to allow creativity to develop, indicated by comments such as ‘sometimes you can’t (be creative); sometimes you do have a set structure of things that you have to follow, and you can’t always be creative, which can sometimes be a bit sad because you want to do something interesting but sometimes you know you have to follow a set structure for an assignment or something’. The students provided suggestions on how their learning environments could support creativity. The students felt that AI could help develop their creativity by encouraging independent thinking and creating opportunities to be creative, such as encouraging ‘new ways to approach different situations’. Another student mentioned, ‘Also, if you’re trying to make a robot move down a path or something, sometimes it’s going to bump into things and it’s going to, you know, go a bit wonky, so you’ve got to think out of the box and you, hang on a second, what’s going wrong here and then backtrack kind of thing, thinking in a different mindset, I guess, to how you usually think.’. 

The students think AI can assist creativity when asked to deepen their thoughts in their learning. It is suggested that schools adopt opportunities for students to engage with creativity and AI as the students desire to engage in these activities. 

### 3.5. Theoretical and Practical Contribution (From 4C to 4AI)

The students’ perceptions of AI varied; those more comfortable with AI had a more comprehensive understanding of the concept. This is in line with the research on trust with AI research (Ashoori and Weisz 2019). Similarly, those who accurately defined creativity and valued the competency tended to think AI could never match human creativity. However, what was notable was that, when students were asked to define AI, they had a very limited understanding of the concept and tended to view AI as general AI or Artificial Superintelligence. The students had experienced an intensive programme using narrow AI, so it was surprising that they did not acknowledge this. Adopting a 4C approach to these results, we propose that the students do not value what we have termed ‘everyday-AI’ (a combination of mini-c and little-c).

It is proposed that the effective integration of AI into classrooms must address the misconceptions students may have about AI. By extending the 4C theory of creativity, we propose a ‘4AI model of Artificial Intelligence’. Following the same principles of the 4C model, we suggest mini-AI, little-AI, Big-AI and legendary-AI. Students described an evident appreciation of Big and legendary AI but did not appear to appreciate the mini or little AI (despite the AI tool being created to support mini-c and little-c). Drawing analogies with the 4C theory of creativity, we propose that thinking about four aspects of AI, perhaps as a ‘4AI Model of Artificial Intelligence in Education’ may be useful. Therefore, educators should focus on this aspect as it is unlikely that Big- or legendary-AI will be as frequently experienced by students in the same way that children are more likely to experience mini-c and little-c. This could include explaining the myths and misconceptions of AI and encouraging students to look for and appreciate examples of mini- or little-AI in their everyday lives. There is also the suggestion that, as with creativity, where there is teaching with creativity, for creativity, and about creativity, there should be teaching for AI, with AI, and about AI. Within these three domains, mini- and little-AI can be explored. It is proposed that students would increase their realistic understandings of AI over time, and some of the issues raised by the students who participated in this programme could be minimised. 

### 3.6. Future Research

This study investigated student perceptions of AI and creativity and has proposed a 4AI model of creativity and AI. Future research could establish this model through both qualitative and quantitative methods. Quantitively, AI-based tasks could be employed in classrooms, delineating mini-AI (perhaps around personalized feedback in learning) versus little-AI. Furthermore, this model could be compared against pre- to post-measures of creativity. Further qualitative work could explore broader perceptions of everyday AI in children and adolescents. Finally, future research should focus on increasing students’ limited views of AI to incorporate more of what AI entails and how widely it permeates society and their learning environments (Yufeia et al. 2020).

### 3.7. Limitations

This study has several limitations. First, this study was limited to secondary school students in South Australia, Australia. Further research should examine and compare K-12 students’ perceptions from other countries and demographics. Secondly, the students reported issues with the AI system effectively working every time they used it. These issues may have contributed to some poorer attitudes for students, if this was their first experience working with AI. Thirdly, whilst the interviews provided rich and in-depth insights into student perceptions, more empirical attitude measures could have been used, which would have provided further insights. 

## 4. Conclusions

The interviews highlighted that the students view the relationship between AI and creativity from four key concepts: social, affective, technological and learning factors. Most of the students reported that, although AI could never match human creativity, AI could certainly help them develop their creativity. A 4AI model of Artificial Intelligence has been proposed to help educators support mini-AI and little-AI experiences, which the findings show was overlooked by the students, despite these being the core of the programme they had experienced. Future research could focus on using AI to address the concerns students mentioned and be used to enhance their creativity. 

## Data Availability

Restrictions apply to the availability of these data. Data was obtained from students and are available from the authors with the permission of the students.

## Appendix A

**Creativity and Artificial Intelligence—a student perspective**

Interview Questions for one-on-one interviews

**Creativity:**

1. What comes to mind when you hear the word ‘creativity’?
2. In what areas of your school life do you see creativity being beneficial?
3. What are the challenges associated with creativity?
4. Are some people more ‘creative’ than others?

I will now move into some questions on artificial intelligence.

5. Do you know what AI is?
6. How comfortable do you feel using AI?
7. How often do you use AI—have you used it before?

**Artificial Intelligence:**

8. What comes to mind when I say the words ‘Artificial Intelligence’?
9. In what areas do you see AI being beneficial?
10. What are the challenges associated with AI?
11. Who can help bring AI into your classroom?
12. What do you think needs to happen to see AI in a classroom?
13. Do you want AI in your classroom?

**Creativity and AI:**

14. What is the relationship between creativity and AI?
15. Can AI be creative?
16. What skills do you think are important for the future of work?
17. How can we support these skills?
18. Can AI ever match human creativity?

Due to nature of the focus groups, we condensed the above 18 questions into 11 questions

**Interview Questions for Focus Groups**

**Artificial Intelligence:**

1. What comes to mind when I say the words ‘Artificial Intelligence’?
2. Do you know what AI is?
3. How comfortable do you feel using AI?
4. How often do you use AI—have you used it before?
5. How do you feel about AI in a collaborative learning environment?
6. Do you want AI in your classroom?
7. What was your experience working with Vianna? What did you like and did not like?

**Creativity and AI:**

8. What comes to mind when you hear the word ‘creativity’
9. Do you think AI can ever match human skills/creativity in the future?
10. What skills do you think are important for the future of work?
11. Bearing your previous discussion in mind, in what ways were you and/or your group creative in this this project?

## Appendix B

**Table A1:** Content units, categories and concepts derived from the qualitative data.

Content Units	Category	Concept
Conversation and lack of awareness	AI Facilitators/Barriers	Social
Student Interest		
Social Intelligence/Social Skills		
Age Barriers		
AI as outlet for creativity		
AI as facilitator for inspiration		
Creativity is a form of self expression		
Comfortable	Comfort level with AI	Affective
Neutral		
Uncomfortable		
Access and use of AI	Perceptions of AI	Technological
Technology Focus		
Advanced Technology		
Automated		
Coding/Programming		
Computers		
Futuristic		
Not human		
Robots		
Technology impeding AI		
Human experience can never compare to machines		
AI provides easy access to information	Impact of school environment	Learning
AI provides global connection		
AI provides idea support		
AI provides learning aid		
AI can increase perspectives		
Creativity helps manage time		
Creativity can increase novel ideas		
Structure of assignments limit opportunities for creativity		
Creative block		
Lack of foundational knowledge to be creative		
Creativity takes time		
It is a risk being creative		
(Lack of) creative experience		
Independent thinker		
Encourage creativity		
Creates opportunities		
Creative Problem Solving		

Table A1 illustrates that the students in the study understood the relationship between creativity and AI in terms of four fundamental dimensions (referred to as ‘concepts’ in the table): social, affective, technological and learning factors.
